# Comammox *Nitrospira* Clade B is the most abundant complete ammonia oxidizer in a dairy pasture soil and inhibited by dicyandiamide and high ammonium concentrations

**DOI:** 10.3389/fmicb.2022.1048735

**Published:** 2022-12-12

**Authors:** Pei-Chun (Lisa) Hsu, Hong J. Di, Keith Cameron, Andriy Podolyan, Henry Chau, Jiafa Luo, Blair Miller, Sam Carrick, Paul Johnstone, Scott Ferguson, Wenhua Wei, Jupei Shen, Limei Zhang, Hongbin Liu, Tongke Zhao, Wenxue Wei, Weixin Ding, Hong Pan, Yimeng Liu, Bowen Li

**Affiliations:** ^1^Centre for Soil and Environmental Research, Lincoln University, Lincoln, New Zealand; ^2^AgResearch, Hamilton, New Zealand; ^3^Lincoln Agritech Ltd, Lincoln University, Lincoln, New Zealand; ^4^Manaaki Whenua – Landcare Research, Lincoln, New Zealand; ^5^The New Zealand Institute for Plant and Food Research, Havelock North, New Zealand; ^6^Department of Microbiology, University of Otago, Dunedin, New Zealand; ^7^Department of Biochemistry, University of Otago, Dunedin, New Zealand; ^8^Fujian Normal University, Fuzhou, China; ^9^Research Center for Eco-Environmental Sciences, Chinese Academy of Sciences, Beijing, China; ^10^Institute of Agricultural Resources and Regional Planning, Chinese Academy of Agricultural Sciences, Beijing, China; ^11^Beijing Academy of Agriculture and Forestry Sciences, Beijing, China; ^12^Institute of Subtropical Agricultural Ecology, Chinese Academy of Sciences, Changsha, China; ^13^Institute of Soil Science, Chinese Academy of Sciences, Nanjing, China; ^14^College of Natural Resources and Environment, Shandong Agricultural University, Taian, China; ^15^Centre for Innovation and Development, Beijing Normal University, Zhuhai, China; ^16^College of Natural Resources and Environment, Hebei Agricultural University, Baoding, China

**Keywords:** comammox *Nitrospira* spp., ammonia-oxidizing bacteria, nitrification, nitrite oxidation, soil microbial ecology, nitrogen cycling

## Abstract

The recent discovery of comammox *Nitrospira*, a complete ammonia oxidizer, capable of completing the nitrification on their own has presented tremendous challenges to our understanding of the nitrification process. There are two divergent clades of comammox *Nitrospira*, Clade A and B. However, their population abundance, community structure and role in ammonia and nitrite oxidation are poorly understood. We conducted a 94-day microcosm study using a grazed dairy pasture soil amended with urea fertilizers, synthetic cow urine, and the nitrification inhibitor, dicyandiamide (DCD), to investigate the growth and community structure of comammox *Nitrospira* spp. We discovered that comammox *Nitrospira* Clade B was two orders of magnitude more abundant than Clade A in this fertile dairy pasture soil and the most abundant subcluster was a distinctive phylogenetic uncultured subcluster Clade B2. We found that comammox *Nitrospira* Clade B might not play a major role in nitrite oxidation compared to the role of canonical *Nitrospira* nitrite-oxidizers, however, comammox *Nitrospira* Clade B is active in nitrification and the growth of comammox *Nitrospira* Clade B was inhibited by a high ammonium concentration (700 kg synthetic urine-N ha^–1^) and the nitrification inhibitor DCD. We concluded that comammox *Nitrospira* Clade B: (1) was the most abundant comammox in the dairy pasture soil; (2) had a low tolerance to ammonium and can be inhibited by DCD; and (3) was not the dominant nitrite-oxidizer in the soil. This is the first study discovering a new subcluster of comammox *Nitrospira* Clade B2 from an agricultural soil.

## Introduction

For over a century, nitrification has been known to involve two separate microorganisms: oxidation of ammonia to nitrite by ammonia oxidizing bacteria (AOB) and/or archaea (AOA), followed by oxidation of nitrite to nitrate by nitrite oxidizers (NOB). The recent discovery of comammox (COMplete AMMonia OXidation) a chemolithoautotrophic bacterium capable of oxidizing ammonia into nitrate, has redefined the century-old paradigm of the two-step nitrification process (Daims et al., 2015; van Kessel et al., 2015; Sakoula et al., 2020). All comammox isolated from enrichment culture and identified to date, belong to the genus *Nitrospira* sublineage II (Palomo et al., 2019; Sakoula et al., 2020). Based on phylogenetic analyses of amino acid of ammonia monooxygenase (AMO), the first enzyme in ammonia oxidation that converts ammonia to hydroxylamine, comammox can be further subclustered into comammox *Nitrospira* Clade A and Clade B (Yu et al., 2018; Palomo et al., 2019; Poghosyan et al., 2019). There are several high-quality comammox *Nitrospira* Clade B-affiliated metagenome-assembled genomes available from environmental studies (Poghosyan et al., 2019; Wang H. et al., 2020), however, so far, all the comammox *Nitrospira* isolates are affiliated with Clade A1, i.e., *Candidatus Nitrospira inopinata* (Daims et al., 2015), Candidatus *Nitrospira* nitrificans, *Candidatus Nitrospira nitrosa* (van Kessel et al., 2015) and *Candidatus Nitrospira kreftii* (Sakoula et al., 2020).

Upon the discovery of the complete ammonia oxidizer comammox *Nitrospira* Clade A, a detailed kinetic analysis was carried out from a pure culture. The study found that both comammox *Nitrospira* Clade A, *Candidatus Nitrospira inopinata*, and *Candidatus Nitrospira kreftii* exhibited high ammonia affinity (Kits et al., 2017; Sakoula et al., 2020) but *Ca. N. kreftii* was found to be partially inhibited by low ammonium concentrations (Sakoula et al., 2020). Li et al. (2019) confirmed the functional role of comammox in nitrification using a ^13^CO_2_-DNA-stable isotope probing study and demonstrated a significant increase of comammox *Nitrospira* Clade A abundance in pasture soil amended with nitrogen fertilizers. In contrast, comammox *Nitrospira* Clade B from forest and paddy soils responded to low nitrogen concentrations supplied from mineralized organic nitrogen but not from added inorganic ammonium (Wang Z. et al., 2019). These findings indicate that ammonium concentration and the source of ammonium play a key role in ammonia oxidation by regulating the activity of comammox *Nitrospira*.

Further studies found comammox *Nitrospira* Clade A in high abundance in dairy pasture soils (Li et al., 2019), vegetable arable soils, forest soils (Li et al., 2020b), acidic soils of a tea field (Takahashi et al., 2020), lake sediments (Shi et al., 2020), salt marches (Bernhard et al., 2021) and plateau wetlands (Yuan et al., 2021), however comammox *Nitrospira* Clade B was absent in all the samples from these studies. This indicated a potential niche separation between comammox *Nitrospira* Clade A and Clade B and raised important questions about the adaptation of comammox to a variety of environments, and whether comammox can compete with canonical nitrifiers AOA, AOB, and NOB in agricultural soils with high ammonia fertilizer inputs. It is known that AOA are mostly found in oligotrophic habitats and AOB are known to dominate eutrophic environments (Verhamme et al., 2011; Herber et al., 2020; Wang X. et al., 2020; Zhao et al., 2020; Semedo et al., 2021). However, comammox *Nitrospira* spp. have also been found to be abundant in various environmental habitats but their role in nitrification is not well understood (Yang et al., 2020; Manasa and Mehta, 2021).

Little is known about the relative contributions of both comammox *Nitrospira* Clade A and B in nitrification, especially in terrestrial ecosystems of animal-based agricultural soils supporting intensive farming and with long-term nitrogen fertilizer inputs. A comprehensive understanding of the nitrification process by both comammox sublineages is needed to re-define our knowledge of the complex nitrogen cycle and its implications for nitrogen management in animal-based agricultural systems. It is wildly known that nitrification inhibitor dicyandiamide (DCD) inhibits the growth of AOB probably by deactivating the AMO enzyme although the exact mode of action is not verified (Di and Cameron, 2011). It is not well understood if comammox *Nitrospira* spp. are also susceptible to nitrification inhibition. Li et al. (2020a) used a microcosm study of agricultural soil to demonstrate that at day 28 the growth of comammox *Nitrospira* Clade A was increased with the addition of nitrogen fertilizers but significantly decreased with the addition of nitrification inhibitors 2-chloro-6-(trichloromethyl) pyridine, 3,4-dimethylpyrazole phosphate, allylthiourea, and DCD (Li et al., 2020a). However, comammox *Nitrospira* Clade B was not detected in the microcosm study and thus has not been investigated.

The objective of this study was to investigate the populations of both comammox *Nitrospira* Clade A and B in New Zealand agricultural soils, compare their activity to canonical nitrifiers AOA and AOB, and the inhibitory effect of DCD on both. The *nxrB* gene that encodes for the beta-subunit of nitrite oxidoreductase and is a phylogenetic marker for canonical *Nitrospira* spp. nitrite-oxidizers (*Nitrospira*-like NOB) was also investigated in this study. We hypothesized that (1) Comammox *Nitrospira* Clade B is abundant in New Zealand agricultural soils; (2) both nitrogen concentration and the nitrification inhibitor DCD affected the growth and population dynamic of comammox *Nitrospira* Clade B; and (3) comammox *Nitrospira* Clade B is a significant nitrite-oxidizer in agricultural soils. To confirm these hypothesise, gene abundance of *amoA* gene (encodes for ammonia monooxygenase alpha subunit) of AOA, AOB, comammox *Nitrospira* Clade A and B and *nxrB* gene for *Nitrospira*-like NOB were determined with a 94-day microcosm study using real-time quantitative PCR. Community structures were determined by 16S rRNA sequencing and the relative gene abundance of nitrifiers was determined by high-throughput sequencing of the partial-nested PCR of the *amoA* gene region. In this research, the population abundance and community structure of comammox *Nitrospira* spp. in animal-based agriculture soil ecosystems were investigated in depth. This study increased our fundamental knowledge and understanding of the role of comammox *Nitrospira* in microbial nitrogen cycling.

## Materials and methods

### Field sampling

Soil samples were collected from the Lincoln University Research Dairy Farm (LURDF; 43°38′26″S, 172°26′37″E) located in Lincoln, on the South Island of New Zealand. The dairy farm soil is Templeton sandy loam [Typic Immature Pallic soil (Hewitt, 2022); USDA: Udic Haplustept, (Keys to Soil Taxonomy, 2014)]. Soil samples were taken randomly 5 m apart from six locations (0–75 mm), transferred in a plastic bucket to the laboratory and thoroughly mixed. Organic debris, grass roots and loose gravel were removed, and the soil was sieved through a 50 mm sieve. A subsample weighing approximately 100 g was used for chemical properties analysis (Supplementary Table S1).

### Microcosm study

In the microcosm study, 585 g of the soil processed above was placed into polypropylene plastic containers (1-L volume). The containers were sealed with lids with two 1 cm holes allowing for airflow and arranged randomly inside an incubator. The samples were incubated at a constant temperature of 12°C for 94 days to mimic autumn/winter local soil temperatures. This is the period when nitrate leaching, and nitrous oxide emissions are high from these grazed pasture soils. The microcosm study included the following six treatments, each with four replicates: Control; 50 kg urea-N ha^−1^ (U50); 50 kg urea-N ha^−1^ with 10 kg ha^−1^ dicyandiamide (DCD, a nitrification inhibitor in liquid form, U50 + D); 700 kg synthetic urine-N ha^−1^ (S700); 700 kg synthetic urine-N ha^−1^ with 10 kg ha^−1^ DCD (S700 + D); Farm Dairy Effluent (FDE, at 50 kg N ha^−1^). Soil moisture content was maintained at approximately 33% (w/w) from Day 1–72 and increased to approximately 42% from Day 73–94 to simulate a rainfall event during the winter season. For measuring ammonium (NH_4_^+^–N) and nitrate (NO_3_^−^–N) concentrations subsamples were taken on day 0 (before treatments), 1, 7, 15, 30, 44, 58, 86, and 94. Soil Subsamples were also kept at −20°C for the DNA extraction and determination of the total abundance of bacterial 16S rRNA, AOA, AOB, *Nitrospira*-like NOB, comammox *Nitrospira* Clade A and comammox *Nitrospira* Clade B.

For NH_4_^+^–N and NO_3_^−^–N analysis, 5 g of fresh soil were taken from each replicate, and 25 ml of 2 M KCl added. The mixture was shaken on a Ratek Platform Mixer for 1 h, centrifuged for 10 min, then filtered through Whatman No. 41 filter paper before being analyzed by a Flow Injection Analyzer (FIA; FOSS FIA star 5,000 triple channel analyzer; Supplementary Table S3).

### DNA/RNA extraction and cDNA synthesis

Genomic DNA of the dairy farm soil samples (0.25 g fresh weight) were extracted using NucleoSpin^®^ Soil Genomic DNA kit (Macherey-Nagel GmbH & Co. KG, Düren, Germany) according to the manufacturer’s instructions. Soil samples at Day 58 was used for RNA extraction using RNeasy^®^ PowerSoil^®^ Total RNA Kit (QIAGEN GmbH, Hilden, Germany) according to the manufacturer’s instructions. In short, 2 g of fresh soil was extracted using phenol/chloroform/isoamyl alcohol mixture (Sigma-Aldrich, St. Louis, MO, United States) and final RNA pellets were resuspened in 30 μl of elution buffer. Resultant RNA products were then treated with TURBO^™^ DNase (InvitrogenTM Thermo Fisher Scientific, Waltham, MA, United States) twice according to the manufacturer’s instructions prior to cDNA synthesis using SuperScript IV One-Step RT-PCR I (InvitrogenTM Thermo Fisher Scientific).

### Cloning and sequencing

Amplification of Comammox *Nitrospira* Clade A and Clade B *amoA* and *nxrB* genes was carried out by PCR from gDNA of LURDF soil sample using Platinum^®^ *Taq* DNA Polymerase, High Fidelity (Invitrogen^™^ Thermo Fisher Scientific, Waltham, MA, United States) to prevent error propagation, ensuring correct replication of the target DNA. The resultant PCR products were purified and concentrated using NucleoSpin^®^ Gel and PCR Clean-up kit (Macherey-Nagel GmbH & Co. KG, Düren, Germany) followed by cloning into pGEM^®^-T Easy Vector System (Promega Corporation, Madison, WI, United States) and transformed into *Escherichia coli* JM109 high-efficiency competent cells, according to the manufacturer’s protocols. Plasmid of prominent colonies were extracted using PureLink^™^ HiPure Plasmid Miniprep Kit (Invitrogen^™^ Thermo Fisher Scientific, Waltham, MA, United States) and confirmed with restriction digest using EcoRI-HF restriction enzyme and subsequently subjected to Sanger sequencing using T7 and SP6 primers by ABI Prism 3130xl Genetic Analyser (Applied Biosystems^™^, Waltham, MA, United States). Sequence results were analyzed by MUSCLE alignment (Edgar, 2004) using Geneious Prime^®^ (Biomatters Ltd., Auckland, New Zealand).

### Quantification of gene abundance

DNA extractions from each soil sample were diluted 10-fold with nuclease-free water before qPCR analyses. Primers were used to amplify 16S rRNA, and regions of genes from AOA, AOB, *Nitrospira*-like NOB and comammox are listed in Supplementary Table S2. Primers used for quantifying comammox *amoA* are CA377f/C576r for Clade A and CB377f/C576r for Clade B, as developed by Jiang et al. (2020). Standard curves of each gene were prepared by serial dilutions of plasmid DNA ranging from 10^−1^ to 10^−7^ copies per μl. Reactions were set up by CAS-1200 Robotic liquid handling system (Corbett Life Science), with each reaction consisting of 8 μl of SYBR Premix^®^ Ex Taq^™^ (TaKaRa Holdings INC., Kyoto, Japan), 0.6–1.0 μl of both forward and reverse primers, 1.5 μl of genomic DNA and nuclease-free water to a final volume of 16 μl (Supplementary materials and methods). Real-time qPCR was carried out using a Rotor-Gene^®^ 6000 real-time rotary analyser (Corbett Life Science, BioStrategy, Auckland, New Zealand). Melting curve analysis was performed at the end of each amplification cycle to confirm the reaction specificity. Standard curves covering seven orders of magnitude, with *R^2^* value ranging between 0.995 and 0.999, and efficiencies of 98%–105%, were obtained.

### Partial nested-PCR and next generation sequencing

The *amoA* genes for both comammox *Nitrospira* Clade A and Clade B were amplified using the partial nested-PCR, as described by Xia et al. (2018) using Platinum^®^ Taq DNA Polymerase, High Fidelity. First round PCR was carried out by A189Y and C576r primer pair at 52°C annealing temperature and CA209f and C576r-barcoded (with 12-nt barcodes) were used for the second round PCR at 49°C (Supplementary Table S2). The final PCR products was purified as described above and PCR samples were pooled together at equal concentration. A sequencing library was constructed by using NEBNext Ultra II DNA Library Prep Kit (New England Biolabs, England) following the manufacturer’s instructions. The genomic DNA fragments were selected with sample purification beads then end polished, A-tailed, and ligated with the full-length adapter. Libraries are sequenced by Illumina NovaSeq PE250 platform with paired-end sequencing strategy through Novogene International Pte. Ltd. (Supplementary Table S4).

### Data and statistical analysis

Raw Illumina reads were paired followed by quality trimming with BBDuk for any remaining Illumine adaptors and remove reads that are less than 100 bp. The quality of raw data was also assessed using FastQC, and low-quality sequences were excluded from downstream analysis. Data normalization was performed by BBNorm resulting even coverage distribution of high-depth area. Forward and reverse reads were then merged by BBMerge, followed by extracting sequences between 400 and 440 bp. Chimeric reads were removed by USEARCH using the UCHIME algorism, and the remaining sequences were extracted and grouped together according to 12-nt barcodes (Edgar, 2004). The 12-nt barcodes were trimmed and deionized before Amplicon sequence variants (ASVs) were identified at the 100% similarity level using DADA2 algorithm and were BLASTN against sequences from the National Center for Biotechnology Information (NCBI) database. Sequences with less than 90% identify value and relative abundances less than 0.05% were excluded from any downstream analyses. Taxonomy information was confirmed by both DNA and amino acid-based phylogenetic trees constructed using ASVs sequences and reference sequences from the NCBI database using the Jukes-Cantor neighbor-joining method by Geneious Tree Builder, with a bootstrapping with 1,000 replicates at >60% supported threshold.

Statistical analysis was conducted using both Genstate (version 21.1.0.25568, VSN International Ltd.) and R (version 4.0.2) software. To compare soil NH_4_^+^–N and NO_3_^−^–N concentration and absolute gene quantifications of each treatment one-way analysis of variance (ANOVA) was conducted followed by Tukey’s Honest Significant Difference (HSD). To assess the community similarity, the relationships between the relative abundances of AOB and comammox *Nitrospira* Clade B were estimated by Principal Component Analysis (PCA) using “Factoextra” R Package. Kruskal-Wallis tests were also performed in R to determine differences in gene abundance and the diversity index among samples. Pearson and Spearman correlations were used to determine the relationships between the abundance of *amoA* genes of *Nitrosospira* spp., *Nitrosomonas spp*., and comammox *Nitrospira* Clade B using the “corrplot” package in R (Supplementary Figures S1, S2).

## Results

### Nitrogen dynamics over the 94-day microcosm study

**Figure 1:**
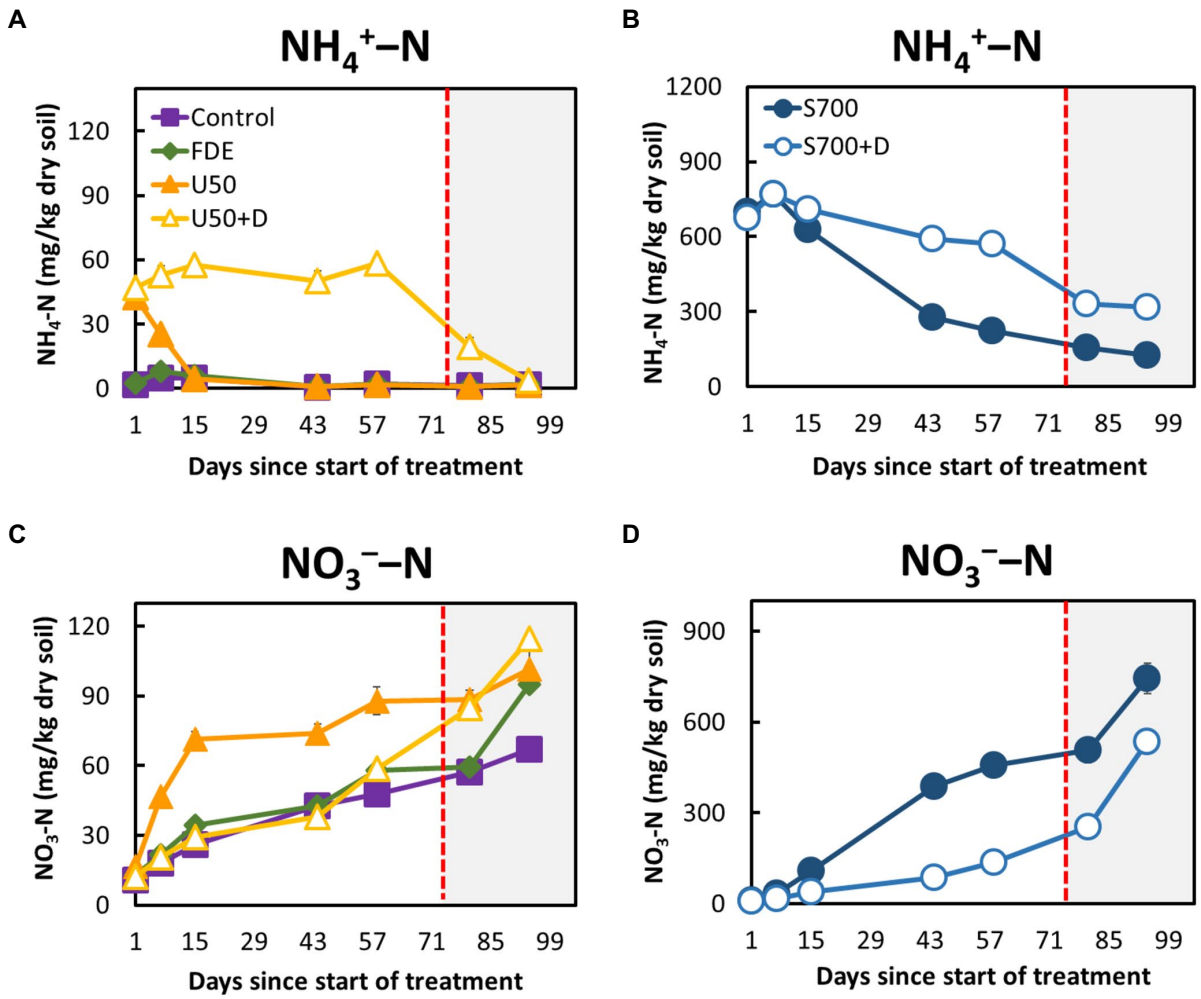
Soil ammonium and nitrate dynamics during the 94-day microcosm study. **(A)** Ammonium–N concentrations in the control, FDE, U50, and U50 + D treatments. **(B)** Ammonium–N concentrations in the synthetic urine treatments, S700 and S700 + D. **(C)** Nitrate–N concentrations in the control, FDE, U50, and U50 + D treatments. **(D)** Nitrate–N concentrations in the synthetic urine treatments (S700 and S700 + D). The unshaded area (Day 1─72) indicates soil moisture content at approximately 33%, and the shaded area (Day 73─94) indicates soil moisture content at approximately 42%. Error bars indicate standard errors of the mean.

The soil NH_4_^+^–N concentrations of control and FDE reduced rapidly from Day 7 to Day 15, then remained relatively unchanged until Day 94 (Figure 1A). The NH_4_^+^–N concentration in the U50 treatment reduced rapidly from Day 1 to Day 15 (42.55 ± 2.09 mg kg^−1^ dry soil to 4.70 ± 0.62 mg kg^−1^ dry soil; Figure 1A). In contrast, the NH_4_^+^–N concentrations in the U50 + D treatment remained unchanged from Day 1 to Day 58 (47.12 ± 2.13 to 58.53 ± 4.46 mg kg^−1^ dry soil), but a sudden reduction was observed when soil moisture content increased to 42% at day 86 (19.50 ± 3.55 mg kg^−1^ dry soil). NH_4_^+^–N concentrations in the S700 + D treatment, containing the nitrification inhibitor DCD, remained between 674.69 ± 45.30 and 570.11 ± 7.27 mg kg^−1^ dry soil over the course of 58 days (Figure 1B), and the NH_4_^+^–N concentrations were greatly reduced at 42% soil moisture content. The NH_4_^+^–N concentrations in the S700 without DCD treatment decreased significantly (*p* < 0.001; Figure 1B).

The NO_3_^−^–N concentrations in the control, FDE and U50 + D treatments increased slowly from Day 1 to Day 58 but increased rapidly when the soil moisture content increased from day 80 to 94 (Figure 1C). A similar trend was observed in the treatment’s synthetic urine treatments (S700 and S700 + D). In the S700 treatment, the NO_3_^−^–N concentration increased rapidly, and was significantly higher than that in the s700 + D treatment (*p* < 0.001; Figure 1D). The addition of DCD effectively reduced the NO_3_^−^–N concentrations in both U50 + D and S700 + D treatments at 33% soil moisture content, but in the U50 + D treatment, the nitrification inhibition effect by DCD was diminished at the high soil moisture content (Figure 1C).

### Comammox *Nitrospira* Clade B is sensitive to ammonium

**Figure 2:**
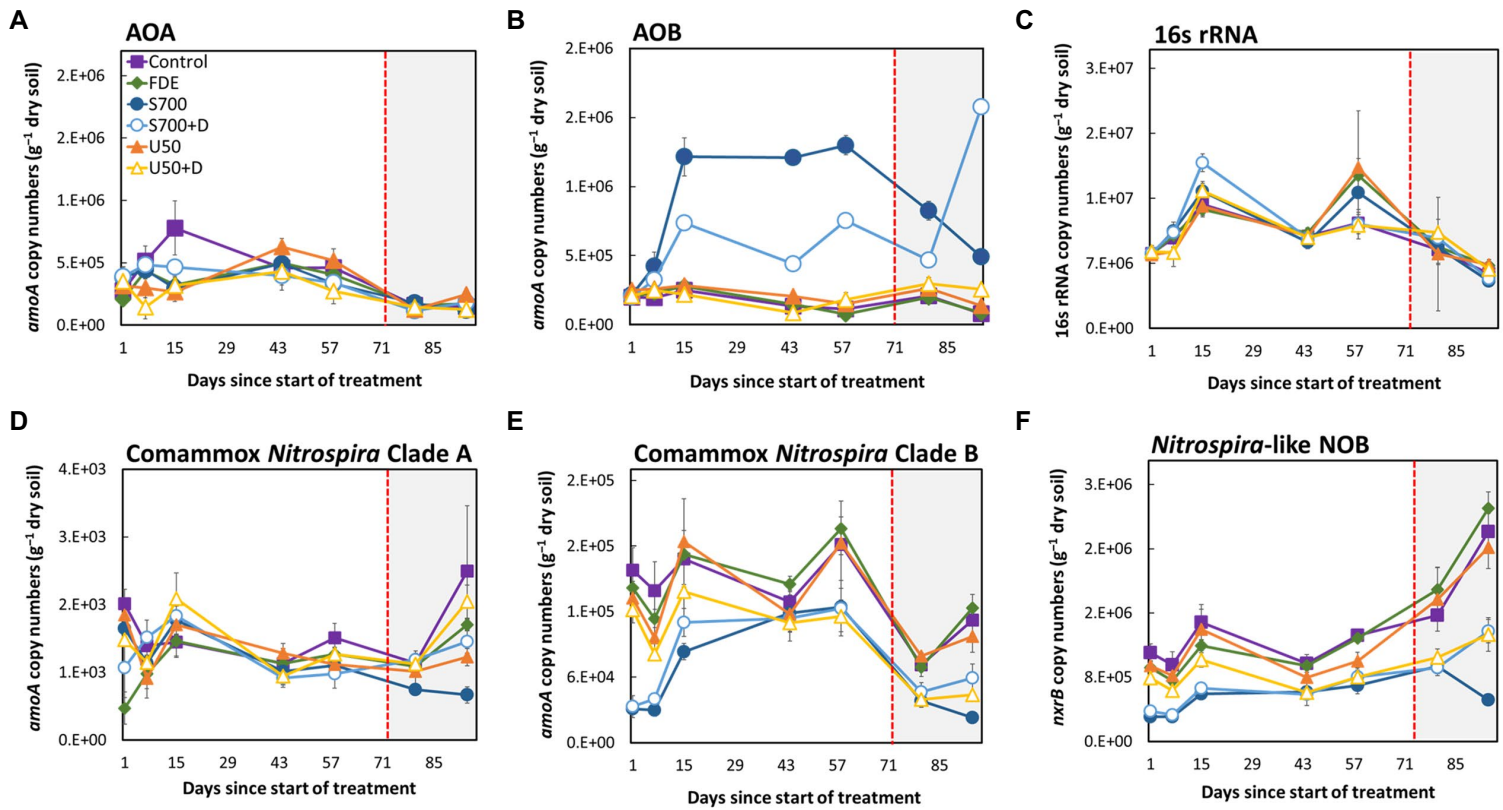
Microbial population dynamics of the 94-days microcosm study. The *amoA* gene abundance of: AOA **(A)**, AOB **(B)**, 16S rRNA (total bacteria) **(C)**, comammox *Nitrospira* Clade A **(D)**, comammox *Nitrospira* Clade B **(E)**, and *Nitrospira*-like NOB **(F)**. The unshaded area (Day 1–72) indicates soil moisture content at approximately 33%, and the shaded area (Day 73–94) indicates soil moisture at approximately 42%. Error bars indicate the standard error of the mean for all sample replicates (*n* = 4).

The abundance of total bacteria, AOA, AOB, comammox *Nitrospira* Clade A, comammox *Nitrospira* Clade B, and NOB in each treatment over the 94-day period is shown in Figure 2. The copy number of AOA *amoA* did not change significantly between all treatments over the 94-day period (Figure 2A), but the copy number of AOB *amoA* increased significantly with the S700 treatment, from 2.14 × 10^5^ copies g^−1^ of dry soil (Day 1) to 1.22 × 10^6^ copies g^−1^ of dry soil (Day 15; Figure 2B, *p* < 0.001). The addition of nitrification inhibitor DCD inhibited the overall growth of AOB significantly compared to S700 at 33% soil moisture content (*p* < 0.001), but the inhibition effect decreased at the 42% soil moisture content (Figure 2B). The total bacterial abundance showed fluctuation during the experimental period, and is decreased by approximately 25%, but no significant differences were observed between treatments (Figure 2C). The abundance of comammox *Nitrospira* Clade A remained largely unchanged in all treatments (Figure 2D), but the growth of the comammox *Nitrospira* Clade B population varied over time, and showed stronger treatment effects (Figure 2E). Results showed that, the addition of synthetic urine-N substrate on its own (S700) and with DCD (S700 + DCD) reduced the abundance of comammox *Nitrospira* Clade B the day after the initial application by 19.7 and 21.0%, respectively, when compared with the untreated control (Figure 2E, *p* < 0.001), but by Day 15 the population in both treatments had increased. The population dynamics of comammox *Nitrospira* Clade B showed no statistical significant difference at Day 44 compared to Day 1, and there are large variations amongst different treatments on Day 58. A similar growth pattern was observed for the total *Nitrospira*-like NOB population at 33% soil moisture content (*nxrB* gene copy number, Figure 2F). By increasing the soil moisture content to 42%, the abundance of *Nitrospira*-like NOB increased dramatically under all except the S700 treatment by Day 94. In contrast, comammox *Nitrospira* Clade B showed a significant decrease in *amoA* gene copy numbers when the soil moisture content was increased to 42% (Figure 2E). Overall, there was a significant reduction in comammox *Nitrospira* Clade B population growth for U50 + D treatment compared with U50 (the same treatment without DCD) at Day 94 (*p* < 0.001), but there was no difference in population growth between the S700 treatments with or without nitrification inhibitor DCD.

### Transcription of comammox *Nitrospira* Clade B *amoA* is inhibited by nitrification inhibitor dicyandiamide

**Figure 3:**
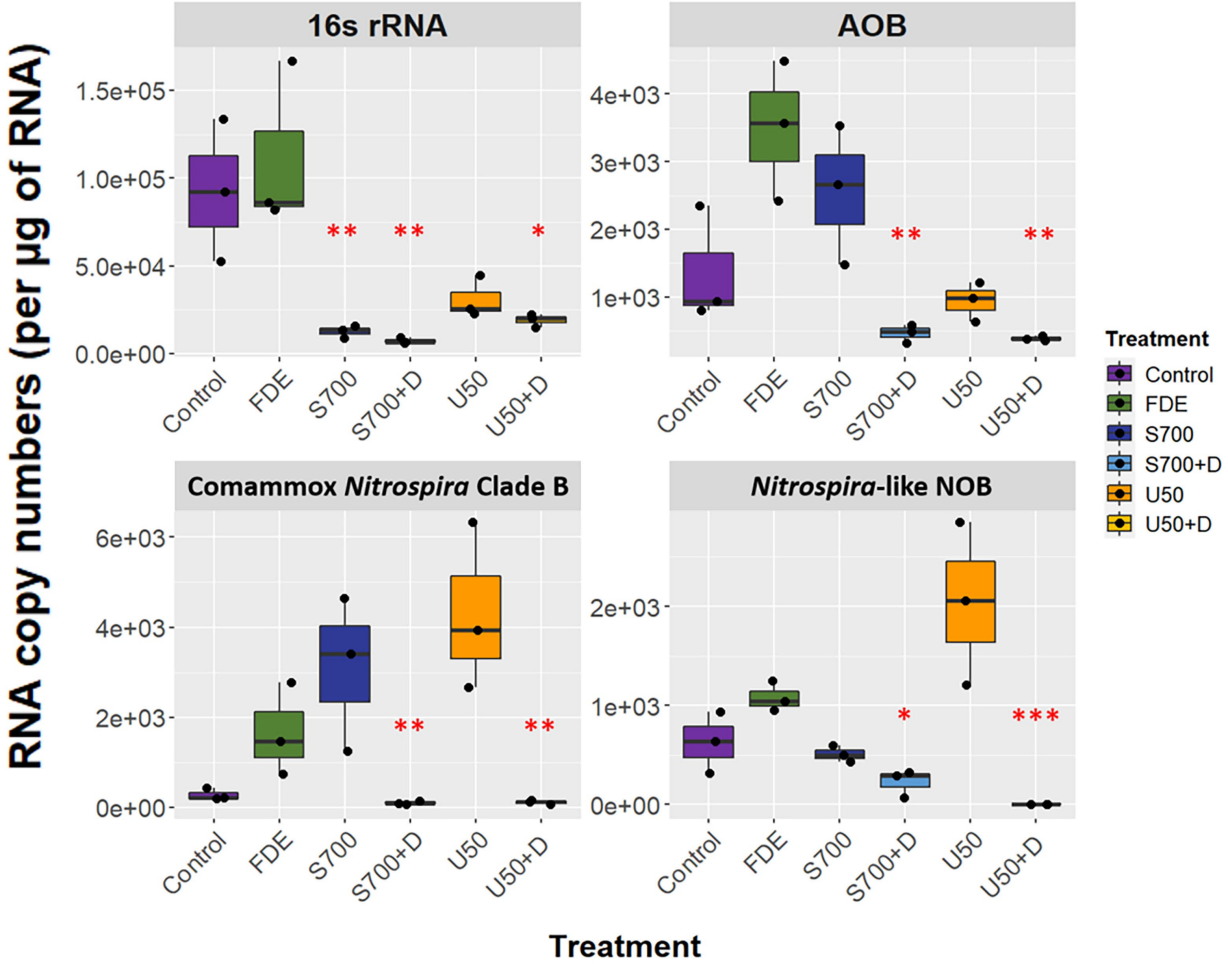
Functional gene expression of the microcosm study at day 58. RNA copy numbers of 16S rRNA, *amoA* of AOB and comammox *Nitrospira* Clade B, and *nxrB* of *Nitrospira*-like NOB at day 58 of the macrocosm study (*n* = 3). Box plot whiskers bar indicates variabilities of maximum upper and lower quartiles. One-way analysis of variance (ANOVA) was used to calculate significant differences between treatments (*p < 0.05, **p < 0.01, ***p < 0.001).

RNA was extracted from each soil sample at Day 58, allowing soil microorganisms to grow over the microcosm study. The qPCR analysis showed that the expression of bacterial 16S rRNA was significantly higher in the control and FDE treatments than in the other treatments, while there was no difference in bacterial 16S rRNA between the S700 and U50 treatments, with or without nitrification inhibitor DCD (*p* > 0.05; Figure 3). The expression level of AOB *amoA* gene was 2.56 ± 0.59 × 10^3^ copies μg^−1^ RNA for S700 treatments and was reduced significantly to 4.69 ± 0.77 × 10^2^ copies μg^−1^ RNA for the S700 with DCD treatment (*p* < 0.001). Similar trends were observed with the U50 treatments, although the expression of AOB *amoA* gene was lower than seen in the S700 treatments (*p* < 0.01). The expression levels of comammox *Nitrospira* Clade B *amoA* between S700 and U50 treatments showed no significant difference, 3.10 ± 0.99 × 10^3^ copies μg^−1^ RNA and 4.31 ± 1.07 × 10^3^ copies μg^−1^ RNA, respectively (*p* > 0.05), but the expression levels in treatments with additional DCD were greatly reduced (*p* < 0.001; Figure 3). The *nxrB* gene for the U50 treatment showed a different trend to those in the control and other treatments, with elevated gene expression (2.04 ± 0.47 × 10^3^ copies μg^−1^ RNA; *p* < 0.001). Neither the *amoA* gene of AOA nor comammox *Nitrospira* Clade A were detected by qPCR at Day 58.

### Phylogenies of AmoA revealed a distinctive subcluster of comammox *Nitrospira* Clade B2

**Figure 4:**
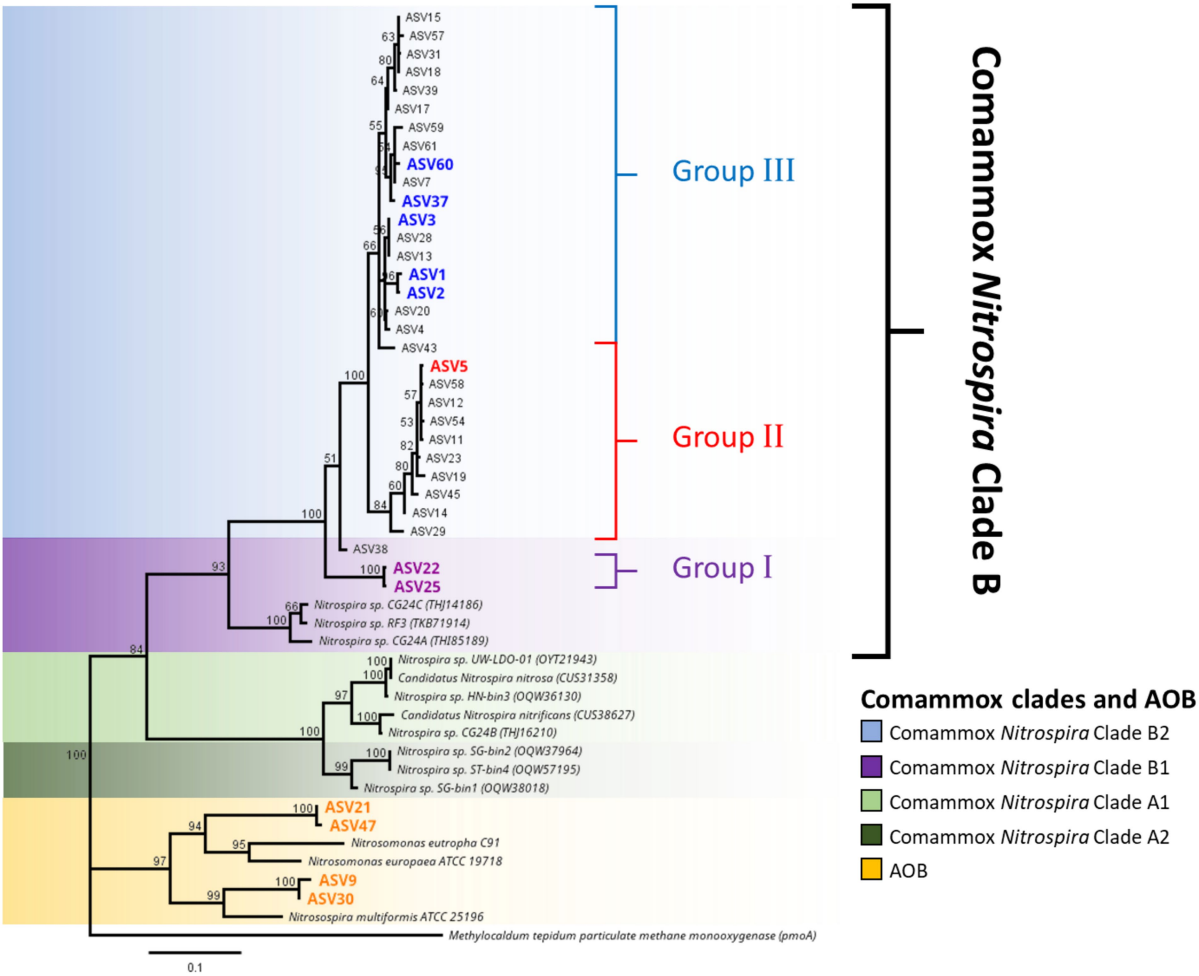
Phylogeny of ammonia oxidizing bacteria and comammox *Nitrospira amoA* sequences. Phylogenetic analysis of comammox *Nitrospira amoA* from high-throughput sequencing was carried out using maximum likelihood alignment analysis. The genetic distance was calculated using the Jukes-Cantor model and the tree was constructed using the neighbor-joining method with over 60% supported threshold. The tree is to scale, and pairwise distances are indicated on the scale bar. Highlighted ASV indicated the most abundant ASVs from each commamox clades and AOB subclasses. Particulate methane monoxygenase (*pmoA*) nucleotide sequence from *Methylocaldum tepidum* was used as the outer group.

**Figure 5:**
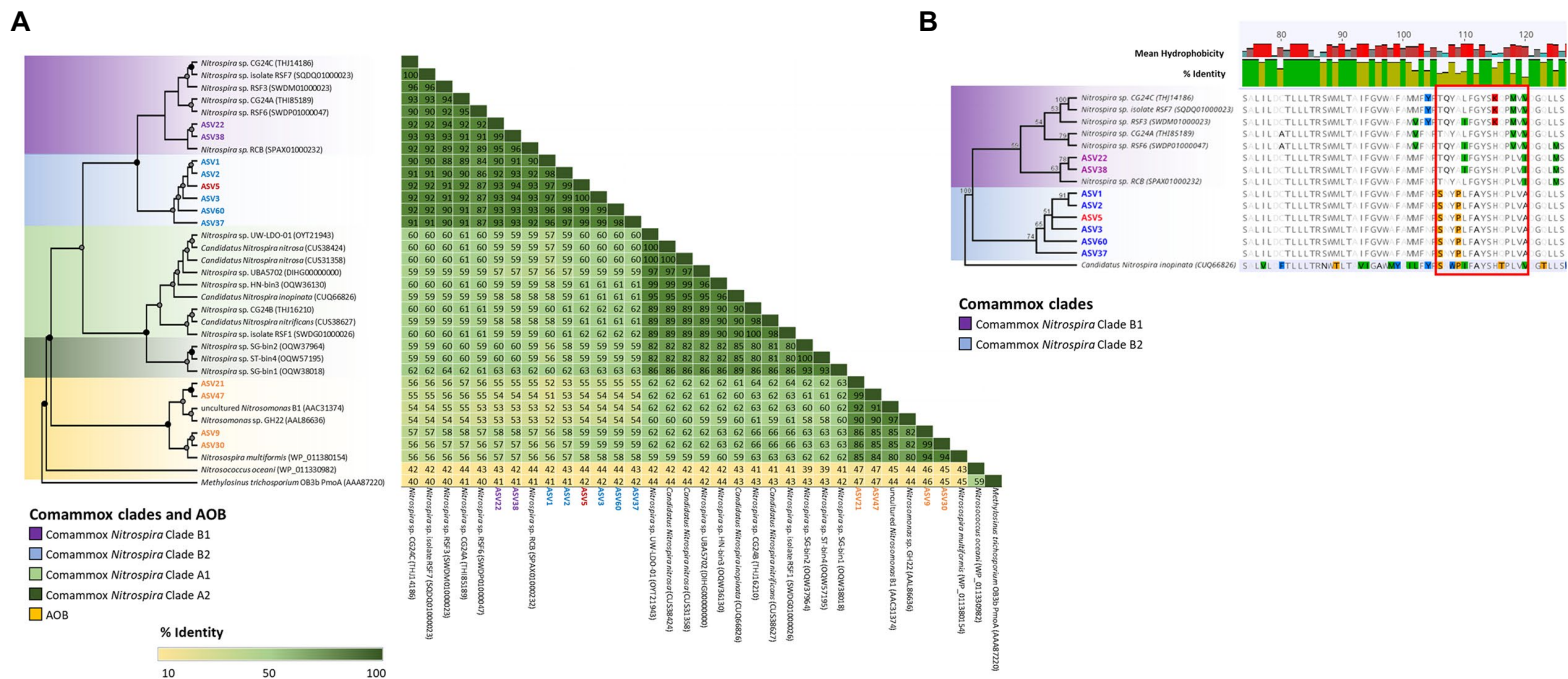
Phylogenetic analysis of comammox *Nitrospira* and ammonia oxidizing microorganisms AmoA sequences. The maximum likelihood tree was constructed using protein alignment of 133 aa sequences **(A)**. Comammox *Nitrospira* clades and ammonia oxidizing bacteria (AOB) are indicated with colored boxes. Bootstrap support values of 100% are indicated with black circles, and ≥60% are indicate with a grey circle. The tree branches were transformed and drawn proportionally **(A)**. Highlighted ASV indicated the most abundant ASVs from each commamox clades and AOB subclasses. *Methylosinus trichosporium* OB3b PmoA (AAA87220) was used as the outer group. Detailed protein alignment is shown from residue 74–126 **(B)** where red boxed area indicates high amino acid variables between comammox *Nitrospira* Clade B1 and B2 (residue 106–120). *Candidatus Nitrospira inopinata* (CUQ66826) was used as an outer group with 100% bootstrap value.

High-throughput sequencing of the *amoA* gene was performed using the partial nested-PCR as described by Xia et al. (2018). After trimming, removing of adapters and barcodes, 400 bp sequences of *amoA* were obtained. Of the more than 180 ASVs, 61 were over 0.05% of the total relative abundance were selected for downstream analysis. Based on the nucleotide BLASTN analysis, 25 ASVs were classified as *Nitrosospira* spp., 3 ASVs are *Nitrosomonas* spp. and 33 ASVs were comammox *Nitrospira* Clade B. There were no comammox *Nitrospira* Clade A obtained from the partial nested-PCR. Based on the phylogenetic analysis of nucleotide sequences, there were three distinctive groups of comammox *Nitrospira* Clade B (Figure 4). Group I consisted of ASV22 and ASV25 and had high nucleotide GC% (52.8% and 52.5% respectively). The remainder of comammox *Nitrospira* clade B were divided into two distinctive groups divergent from ASV38 (Group II and Group III) and were found low in GC% (49.9%–51.0%, an average of 50.7%; Figure 4). The partial *amoA* sequences from partial nested-PCR were translated into 133 amino acids which revealed two unique groups of comammox *Nitrospira* Clade B, where AmoA of ASV3 (50.5% GC) shared 93.2% amino-acid identity with ASV22 (52.8% GC) and 87.2% with comammox Clade B *Nitrospira* sp. RSF6 (52.5% GC, SWDP01000047; Figure 5A). Nucleotide sequence with high GC% (ASV22 and ASV25) was hereafter classified as comammox *Nitrospira* Clade B1, and sequences with low GC% (i.e., ASV3) classified as comammox *Nitrospira* Clade B2. All ASVs from Group II and III were categorize as Clade B2 due to high amino-acid conservation (above 97%). Phylogenetic analysis showed region from Thr106 to Ile120 (ASV22, 15 aa) and Ser106 to Ala120 (ASV3) is the most diverse region between Clade B1 and B2, respectively, which represented Thr165 to Val179 of *Nitrospira* sp. RSF6 AmoA protein sequence (Figure 5B; Supplementary Figure S3).

### Comammox *Nitrospira* Clade B2 is the dominant subcluster of comammox in agriculture soil

**Figure 6:**
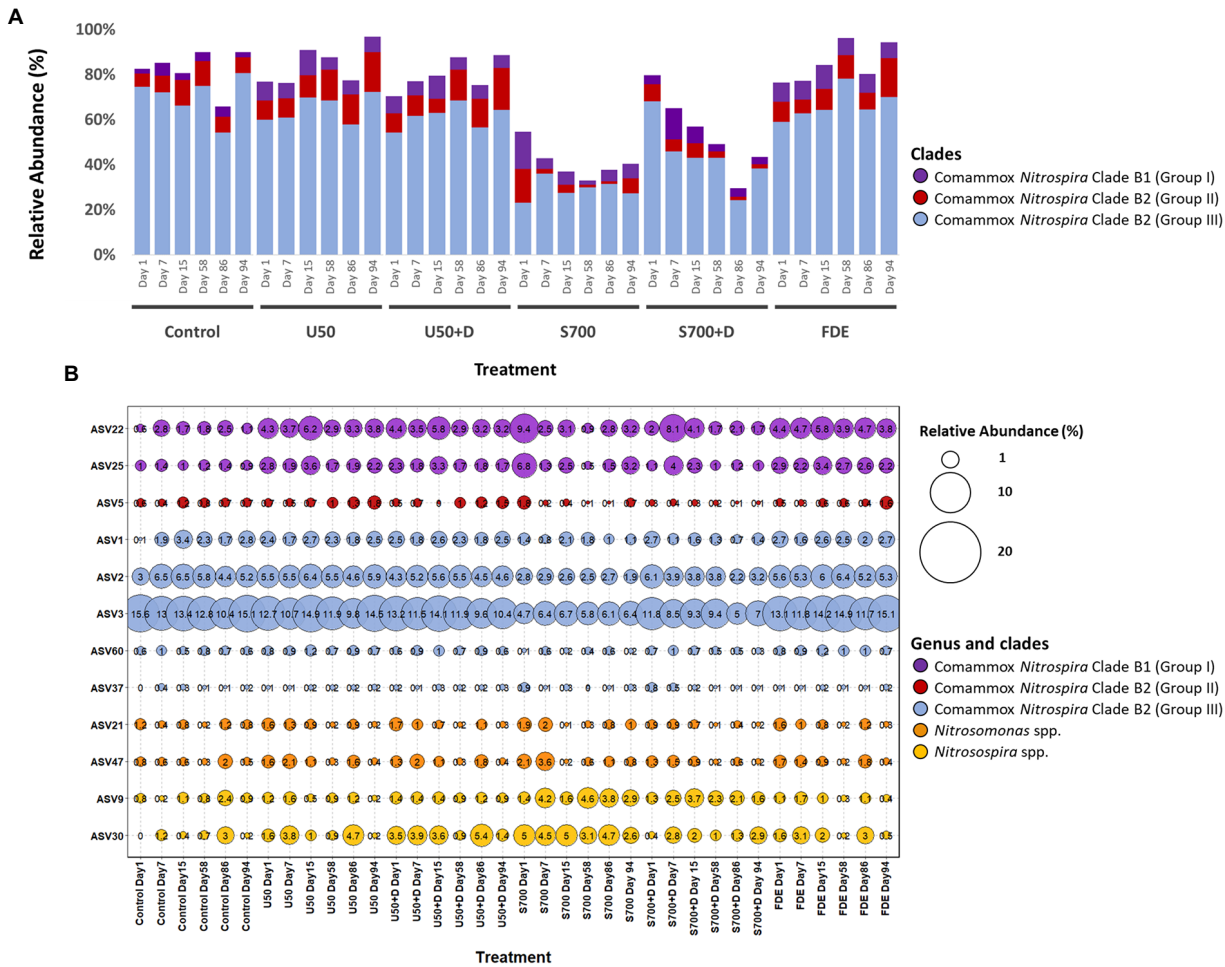
Relative abundance of ammonia oxidizers based on high-throughput sequencing of the *amoA* gene. **(A)** Relative abundance of the total comammox *Nitrospira* Clade B1 (Group I – purple), Clade B2 (Group II – red) and Clade B2 (Group III – blue) of each treatment at Day 1, 7, 15, 58, 86, and 94. **(B)** Relative abundance (percentage in proportion) of selected ASVs of *Nitrosospira* spp., *Nitrosomonas* spp., and comammox *Nitrospira* Clade B1 and B2.

High-throughput sequencing of *amoA* using partial nested-PCR revealed three distinctive groups of *amoA* from comammox *Nitrospira* Clade B, *Nitrosospira* spp. and *Nitrosomoans* spp. Results from the control treatment at Day 1 showed that comammox *Nitrospira* Clade B2 (Group III) represented the majority of the population (74.50%; Figure 6A), whereas the population dropped to 59.99% with U50 treatment, and 23.23% with synthetic urine treatment (700 kg urine-N ha^−1^). Comammox *Nitrospira* Clade B1 (Group I) increased its relative abundance from 2.13% (control treatment at Day 1) to 8.24 and 16.46% (U50 and S700 treatments, respectively). These results indicate both comammox *Nitrospira* Clade B1 (high GC content) and Clade B2 (low GC content) are sensitive to ammonium.

The relative abundance of variant of ASV3 with the control sample at Day 1 was 15.60% and remained relatively unchanged across this study (Figure 6B). A similar finding was also observed with the U50 treatment. However, the relative abundance of ASV3 decreased significantly with S700 and S700 + D treatments compare to the control. A comparable result was observed with other ASVs residing in Clade B2 (Figure 6B). In contrast, comammox *Nitrospira* Clade B1 displayed a different response pattern, where the relative abundance of ASV22 for U50 treatment increased from 4.3% on Day 1 to 6.2% on Day 15. ASV22 also showed large variations with S700 treatment where the relative abundance decreased from 9.4% on Day 1 to 3.1% on Day 15.

**Figure 7:**
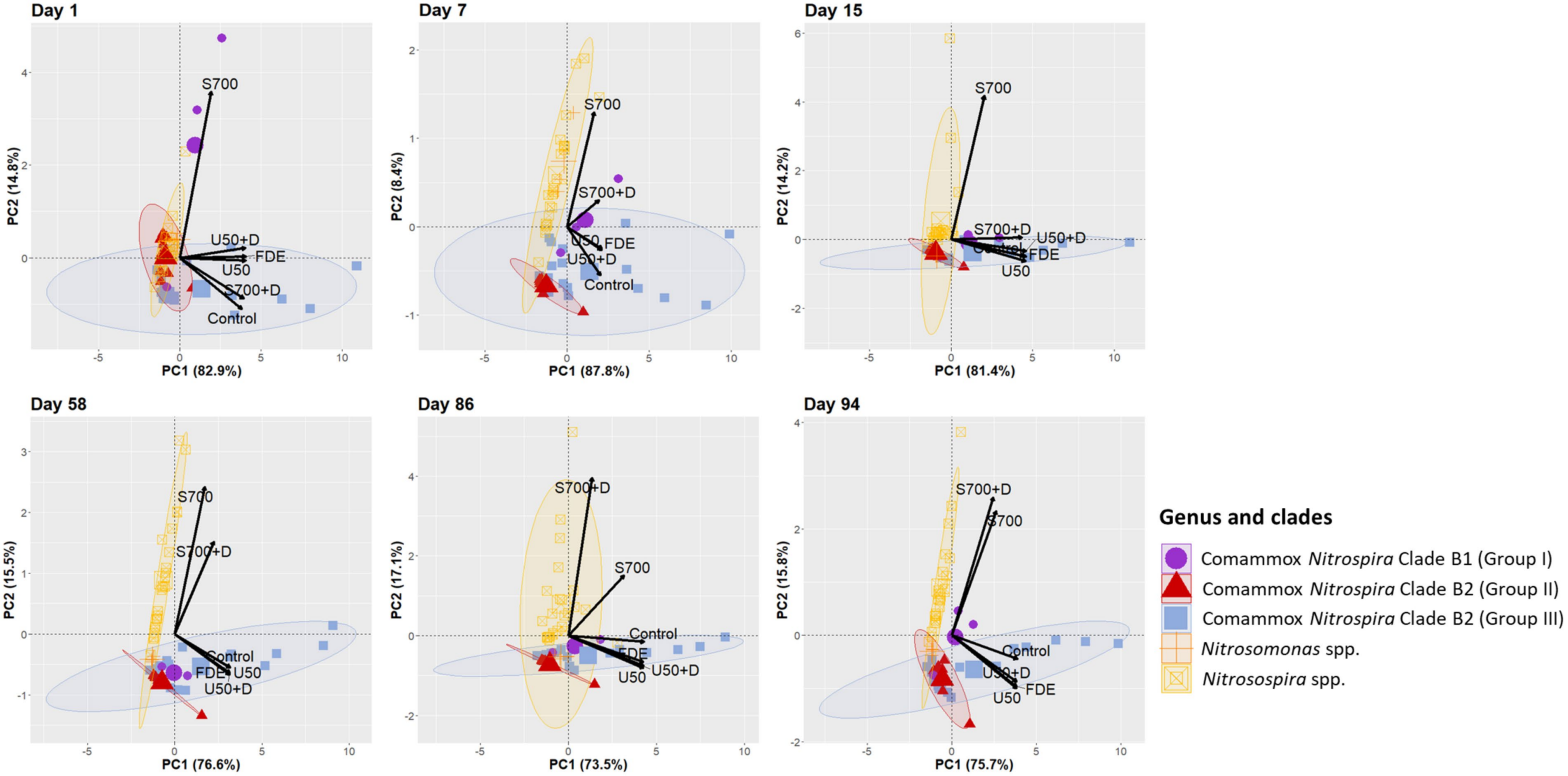
Principal Component Analysis of all ASVs correspond to different treatments. PCA plot based on the relative abundance of ammonia oxidizers *Nitrosospira* spp., *Nitrosomonas* spp., and comammox *Nitrospira* Clade B1 and B2 on Day 1, 7, 15, 58, 86, and 94. Relative abundance of each ASV was calculated by dividing the absolute number of each ASV from the total number of clean paired-end sequenced reads from Illumina sequencing. Ellipses around each ammonia oxidizers group are drawn at 95% confidence level.

Multivariate analysis was performed to explain variations in the responses to treatments from ammonia oxidizer communities. Samples were clustered by *Nitrosomonas* spp., *Nitrosospira* spp., comammox *Nitrospira* Clade B1 and Clade B2 (Group II and Group III; Figure 7). Principal Component Analysis showed that on Day 1 comammox *Nitrospira* Clade B1 (purple circle) was highly associated with the S700 treatment, but less associated from Day 15 onwards, with minimal association to other treatments, apart from a positive association with S700 + D treatment on Day 7. In contrast, the population dynamic of comammox *Nitrospira* Clade B2 (Group III, blue ellipse) was highly associated with low ammonium inputs in the U50, U50 + D and FDE treatment (at 95% confidence level). The treatments with high ammonium concentrations S700 were largely associated with AOB (yellow ellipse). On Day 15, AOB *Nitrosospira* spp. (yellow ellipse) showed a high correlation with S700 treatment (at 95% confidence level, correlation coefficient = 0.898, *p* = 1.21e^−22^) and was associated with S700 + D treatment at Day 86 and 94 (Figure 7, correlation coefficient = 0.904, *p* = 1.89e^−23^; Supplementary file). In contrast, *Nitrosomoans* spp. (orange cross) showed no strong correlations with any treatments at any time points throughout the study, apart from the S700 treatment at Day 7.

## Discussion

Since the discovery of comammox *Nitrospira* in 2015, our understanding of the two-step nitrification process has improved (Koch et al., 2019; Lu et al., 2020; Vijayan et al., 2021). However, only a few comammox *Nitrospira* spp. have been successfully enriched and most species identified reside in Clade A subclusters. The ecological niche separation of comammox *Nitrospira* Clade A and B found in terrestrial ecosystems highlights the complexity of the ecophysiology of comammox and that the nitrification mechanisms of comammox *Nitrospira* spp. require further investigation (Li et al., 2020b; Lin et al., 2020; Wang H. et al., 2020). This study provided a deeper understanding of the abundance of comammox *Nitrospira* and the community structure of its subclusters found in animal-based agricultural soil. This study revealed that the fertile dairy pasture soil has a high abundance of comammox *Nitrospira* especially the Clade B2 subcluster, whereas comammox *Nitrospira* Clade A was undetectable in the high-throughput sequencing of partial-nested PCR. This observation indicates comammox *Nitrospira* Clade A may have minimal contribution to the overall nitrification process in this nitrogen-rich and fertile animal-based agricultural soil. These observations are in contrast to findings by Xu et al. (2020) who reported that the abundance of comammox *Nitrospira* Clade A was two orders of magnitude higher than that of Clade B. However, their study was carried out on a cropping agricultural soil (vegetable, wheat, rice and fruit; Xu et al., 2020), whereas this study was carried out on animal-based dairy pasture soil.

A thorough literature search found no studies with only comammox *Nitrospira* Clade B existing in soil samples. Therefore, low abundance of Clade A found in this New Zealand dairy pasture soil required further investigation, and specific roles of each Clade in the soil defined. It should be noted that although the comammox *Nitrospira* primers for the partial-nested PCR selected for this study were described as being high coverage (Shi et al., 2020), primer CA209f failed to amplify comammox *Nitrospira* Clade A community, whereas primer CA377f from (Jiang et al., 2020) provided limited detection using qPCR, although both methods used the same reverse primer C576r (Supplementary Table S2). Furthermore, primers established by Pjevac et al. (2017) failed to amplify a defined single band on agarose gel, providing multiple unspecific bands for both Clade A (coma-24F/coma-659R) and Clade B (comaB-244F/comaB659R) from our animal-based agriculture soil. Therefore, the development of effective high coverage primers is urgently required for the successful amplification of comammox *Nitrospira* in agricultural soils.

Both comammox *Ca. N. kreftii* from an enrichment, and *N. inopinata* from a pure culture exhibited high ammonia affinity [K_m(app)_NH3_ ≈ 0.040 ± 0.01 μM and K_m(app)_NH3_ ≈ 0.049 μM, respectively] and are commonly found in low ammonium ecosystems (Daims et al., 2015; Sakoula et al., 2020). These observations suggest comammox *Nitrospira* spp. could be the main driver for microbial nitrification in low ammonium ecosystems that has a competitive advantage at very low ammonia concentrations and high adaptation to oligotrophic environments (Kits et al., 2017; Yang et al., 2020). Sakoula et al. (2020) also observed that *Ca. N. kreftii* exhibited partial inhibition with ammonium concentration at 25 μM, indicating low tolerance to ammonium. A long-term fertilization experiment conducted by Wang J. et al. (2019) using Terminal restriction fragment length polymorphism (T-RFLP) combined with clone-library assays of comammox *amoA* revealed relative abundance of comammox Clade A from a waterloggogenic paddy soil increased significantly with the amount of nitrogen fertilizer added, while comammox *Nitrospira* Clade B decreased (Wang J. et al., 2019). Our study also found that the dairy pasture soil amended with U50 and S700 decreased comammox *Nitrospira* Clade B population 24 h after the treatment by 1.2-fold and 5.1-fold, respectively (Figure 2). The dairy pasture soil amended with S700 + D [dicyandiamide (DCD), a nitrification inhibitor] also reduced the comammox *Nitrospira* Clade B population by 4.8-fold compared to the non-treated control, indicating that the ammonium concentration could be one of the the key factor in regulating comammox growth, especially, in Clade B. Furthermore, high-throughput sequencing revealed that comammox Clade B1 which consist of high nucleotide GC% had a lagging response to S700 treatment compared to Clade B2 (Figure 7), this suggests that Clade B1 may have lower ammonium affinity compared to Clade B2, and Clade B2 is significantly more sensitive to ammonium concentration changes in animal-based agricultural soil. Ammonium sensitivity may be due to different types of ammonium transporters (Amt/Rh-type ammonium transporters). It is known that comammox *Nitrospira* Clade A consist of Rh-type ammonium transporters whereas Clade B consist of Amt-type ammonium transporters which have a higher affinity for ammonia but lower ammonium uptake rates (Fowler et al., 2018; Palomo et al., 2018; Koch et al., 2019; Xu et al., 2020). Intriguingly, it was found that some comammox *Nitrospira* Clade B contains more than one copy of the Amt-type ammonium transporter within genome, which, it is proposed, survives better in fluctuating ammonium environments (Fowler et al., 2018; Koch et al., 2019). This evidence, together with our research results, show a difference in ammonium sensitivities amongst Clade B1 and B2. This may be the driving force behind the selective growth for comammox *Nitrospira* Clade B2 observed in the soils in this study.

It is known that AOA and AOB are generally the nitrifying soil microorganisms that dominate the net nitrification activities in soils with long-term fertilizer input (Shi et al., 2018; Wang J. et al., 2019; Huang et al., 2021). Furthermore, it was found nitrification is driven by AOB rather than AOA in grassland soils and AOB prefers high ammonia substrate whereas AOA prefers a low ammonia environment (Di et al., 2009, 2010). This study shows that the abundance of AOB is approximately 2.5-fold higher than comammox *Nitrospira* Clade B in the control treatment and 14.6-fold higher than in the S700 treatment at Day 58. This suggests that AOB thrives when high ammonium is present which agrees with the previous study (Di et al., 2009). The result is also supported by 16S rRNA gene sequencing of the most abundant ASVs across treatments (Supplementary Figure S4). This study also revealed that the functional transcription of *amoA* of comammox *Nitrospira* Clade B showed no difference when comparing U50 and S700 treatment at Day 58 (Figure 3). However, transcription of *amoA* of comammox *Nitrospira* Clade B was significantly inhibited with the addition of nitrification inhibitor, DCD, to both the U50 and S700 treatments when compared to the control (Figure 3), indirectly confirming the role of *Nitrospira* Clade B in the nitrification process, as DCD is thought to inhibit the activity of the AMO enzyme in ammonia oxidizers (23). Zhao et al. (2020) found that both DCD and DMPP were able to inhibit the activity of comammox *Nitrospira* Clade A in a paddy black alkaline soil (Zhou et al., 2020), however, Clade B was not detected in the soil and therefore the inhibitory effect of Clade B was not investigated. This is contrary to the study conducted by Luchibia et al. (2020) which showed that DMPP did not inhibit both comammox *Nitrospira* Clade A and B from grey vertisol soil. In the current study, reverse transcriptional qPCR indicates that comammox *Nitrospira* Clade B did contribute to the overall nitrification process, but due to lack of representative comammox *Nitrospira* Clade B enrichment and pure culture, the inhibitory effect on Clade B using nitrification inhibitors requires further investigation, specifically transcriptomic sequencing analysis is needed to validate the result.

In this study, canonical *Nitrospira*-like NOB displayed a 5.2─29.3-fold greater abundance than comammox *Nitrospira* Clade B, and that the population dynamic is driven by nitrate levels (Supplementary Figure S5). When the soil moisture content was increased to 42%, which correlates with nitrate accumulation, *Nitrospira*-like NOB abundance increased, while the growth of comammox *Nitrospira* Clade B decreased drastically (Figure 2E). Furthermore, it was found that the growth of Clade B was not driven by nitrate in soil (Supplementary Figure S5). This suggests that comammox *Nitrospira* Clade B may not play a major role in nitrite oxidation and is potentially unable to compete with canonical *Nitrospira*-like NOB for nitrite substrate.

Although two comammox *Nitrospira* KAN-bin2 and *Nitrospira* CTRL-LIN-TMP-bin1 from Clade A encoded for cyanate hydratase (*cynS*) that is closely related to *Ca. Nitrospira* sp. LK70 and canonical NOB, *Nitrospira moscoviensis* (Yang et al., 2020; Wang Y. et al., 2021) but comammox *Nitrospira* spp. consist of high diversity in urea transporters genes and lacks in *cynS*, indicating a considerable genome difference compared to canonical *Nitrospira* NOB (Palomo et al., 2018). The study found that using Ntspa-cynSF/Ntspa-cynSR primers developed by Jiang et al. (2020), *cynS* gene was absent in the tested soil (data not shown). This indicates that not all canonical *Nitrospira* NOB and comammox *Nitrospira* spp. consist of cyanate transporters (CynABD), especially Clade B. This also indicated that the *cynS* gene might not be suitable for determining population growth of *Nitrospira* spp. in agricultural soils.

There have only been a small number of studies investigated comammox *Nitrospira* spp. from agricultural soils. A study conducted by He et al. (2021), who investigated agricultural soils from China found that comammox *Nitrospira* Clade B participates in the nitrification process rather than Clade A (He et al., 2021). This observation agrees with the present study where RNA transcription of comammox *Nitrospira* Clade B *amoA* was observed with amended nitrogen samples, whereas RNA transcription of *Nitrospira* Clade A *amoA* was undetected at Day 58 (Figure 3). However, niche separation was not observed in the He et al. study, instead, Clade A and B were found to co-exist and to be approximately equal in their relative abundance. Liu et al. (2021) demonstrated that Clade B dominated in the plateau area (with higher pH and lower temperature) studied, whereas Clade A outcompeted Clade B in the mountain, foothill and estuarine areas along the Yangtze River (Wang H. et al., 2020). Surprisingly, the community structure of comammox *Nitrospira* were found to either consist of *Nitrospira* Clade A on its own (Li et al., 2020b, 2021; Zhao et al., 2021), or co-occurring with Clade B (Wang J. et al., 2019; Wang X. et al., 2021; Lin et al., 2020, 2022; Sun et al., 2021). This raises an interesting question as to what the key environmental biotic and abiotic factors influence and shape comammox niche separations in New Zealand animal-based agricultural soils. Interestingly, a study conducted by Lin et al. (2020) using a long-term fertilization experiment established since 1988, demonstrated Clade A as the dominant comammox cluster and was co-occurrence with Clade B, but pig manure increased the relative abundance of Clade B significantly along with an increase in soil pH and nutrients (Lin et al., 2020). Moreover, a more recent study conducted using the same soil, found soil treated with phosphate and potassium (PK) increased the relative abundance of comammox *Nitrospira* Clade B2, whereas soil treated with nitrogen, phosphate, and potassium (NPK) with the addition of pig manure increased the abundance of comammox *Nitrospira* Clade B1 (Lin et al., 2022). It was also discovered that comammox *Nitrospira* Clade A was positively associated with pH suggesting that pH may be is a niche defining parameter between comammox *Nitrospira* Clade A and B abundance in terrestrial ecosystems (Xu et al., 2020).

A combination of findings of other research with the results of this study indicates that agricultural management such as cropping or animal-based agriculture, different nitrogen fertilizer applications, soil pH, and the oxygen diffusion in soil together may be the key environmental factors shaping niche separation between comammox *Nitrospira* Clade A and B. Further research will help define the relationship between comammox clades under different land uses, especially between cropping and animal-based agricultural soils, and natural habitats. Furthermore, a representative comammox *Nitrospira* Clade B pure culture is urgently needed to advance our understanding of its contribution to the nitrification process especially regarding nitrite oxidation and its ecology in complex agricultural environments.

## Data availability statement

The original contributions presented in the study are included in the article/Supplementary material, further inquiries can be directed to the corresponding author.

## Author contributions

PCH: investigation, methodology, resource, data curation, formal analysis, software, validation, visualization, and writing—original draft. HD: leader of the project, funding acquisition, project conceptualization, methodology, supervision, and writing—review and editing. KC: conceptualization, supervision, writing—review and editing, and funding acquisition. AP: supervision of experimentation, technical advice, and review and editing. HC, JL, BM, SC, PJ, SF, and WHW: assistance with funding acquisition, and review and editing. JS, LZ, HL, TZ, WXW, WD, HP, YL, and BL: international collaborators, and review and editing. All authors contributed to the article and approved the submitted version.

## Conflict of interest

BM was employed by the company Lincoln Agritech Ltd.

The remaining authors declare that the research was conducted in the absence of any commercial or financial relationships that could be construed as a potential conflict of interest. 

## Publisher’s note

All claims expressed in this article are solely those of the authors and do not necessarily represent those of their affiliated organizations, or those of the publisher, the editors and the reviewers. Any product that may be evaluated in this article, or claim that may be made by its manufacturer, is not guaranteed or endorsed by the publisher.
