# Supplementary material for: Comammox Nitrospira Clade B is the most abundant complete ammonia oxidizer in a dairy pasture soil and inhibited by dicyandiamide and high ammonium concentrations
Source: Front Microbiol. 2022 Dec 12;13:1048735. doi: 10.3389/fmicb.2022.1048735 (PMC9791190; doi:10.3389/fmicb.2022.1048735)
Supplement: Supplementary file 1 [file Data_Sheet_1.docx]

# Supplementary Information for;

***Comammox Nitrospira Clade B is the most abundant complete ammonia oxidizer in a dairy pasture soil and inhibited by dicyandiamide and high ammonium concentrations***

# Authors

Pei-Chun (Lisa) Hsu^1^, Hong J. Di^1*^, Keith Cameron^1^_,_ Andriy Podolyan^1^, Henry Chau^1^, Jiafa Luo^2^, Blair Miller^3^, Sam Carrick^4^, Paul Johnstone^5^, Scott Ferguson^6^, Wenhua Wei^7^, Jupei Shen^8^, Limei Zhang^9^, Hongbin Liu^10^, Tongke Zhao^11^, Wenxue Wei^12^, Weixin Ding^13^, Hong Pan^14^, Yimeng Liu^15^, Bowen Li^16^

^1^Centre for Soil and Environmental Research, Lincoln University, Lincoln, 7647, Christchurch, New Zealand;

^2^ AgResearch, Ruakura, Hamilton, New Zealand;

^3^Lincoln Agritech Ltd, Lincoln University, Christchurch, New Zealand;

^4^Manaaki Whenua - Landcare Research, Lincoln, Canterbury, New Zealand;

^5^ The New Zealand Institute for Plant and Food Research Limited, Hastings, Havelock North 4172, New Zealand;

^6^Department of Microbiology, University of Otago, Dunedin, New Zealand;

^7^ Department of Biochemistry, University of Otago, Dunedin, New Zealand.

^8^Fujian Normal University, Fuzhou, China;

^9^Research Centre for Eco-environmental Science, Chinese Academy of Sciences, Beijing, China;

^10^Institute of Agricultural Resources and Regional Planning, Chinese Academy of Agricultural Sciences, Beijing, China;

^11^Beijing Academy of Agriculture and Forestry Sciences, Beijing, China;

^12^Institute of Subtropical Agricultural Ecology, Chinese Academy of Sciences, Changsha, China;

^13^Institute of Soil Science, Chinese Academy of Sciences, Nanjing, China;

^14^College of Natural Resources and Environment, Shandong Agricultural University, China;

^15^Centre for Innovation and Development, Beijing Normal University, China;

^16^College of Natural Resources and Environment, Hebei Agricultural University.

*Corresponding author: Hong J Di, E-mail: [Hong.Di@lincoln.ac.nz](mailto:Hong.Di@lincoln.ac.nz)

**Supplemental materials and methods**

## *Field sampling*

Soil samples were collected from a farm that has a mean annual maximum air temperature of 32°C and minimum temperature of 4°C, and receives an average annual rainfall of 666 mm, and an average of 2040 sunshine hours per annum (<http://www.siddc.org.nz/lu-dairy-farm>). The soil receives approximately 200 kg N ha^−1^ of urea per annum, split into about 8 applications through the growing season (August to May) with no supplementary dairy effluent applied. Additional nutrients such as phosphate, sulphate, and magnesium are applied as required to achieve optimal soil fertility. Irrigation is applied during the summer months to supplement natural rainfall. A subsample weighing approximately 100 g was used for chemical properties analysis (Table S1).

## *DNA/RNA extraction and cDNA synthesis*

The quality and concentration of extracted gDNA were measured by NanoDrop ND2000c spectrophotometer (NanoDrop Technologies, Thermo Scientific, MA, USA) and Quanti-iT^TM^ dsDNA BR assay kit using a Qubit fluorometer (Life Technologies, Auckland, New Zealand). Prior to the cDNA synthesis, the final RNA concentration was measured by the Quant-iT™ RNA Assay Kits (Invitrogen^TM^ Thermo Fisher Scientific) using a Qubit fluorometer.

### **Quantitative PCR**

Each 16 µL PCR contains of 8 µL of SYBR Premix^®^ Ex Taq^™^ (TaKaRa Holdings INC., Kyoto, Japan), 0.6–1.0µL of both forward and reverse primers, 1.5 µL of genomic DNA and nuclease-free water. Primers were used at a final concentration of 400 nM for AOA and AOB, and 500 nM for 16S rRNA, commamox *Nitrospira* Clade A & B and *Nitrospira*-like NOB *nxrB* gene. Conditions for qPCR of AOA, AOB, and commamox *Nitrospira* Clade A *amoA* genes were 94°C for 2 mins, followed by 40 cycles of 94°C for 20 s, 55°C for 30 s, 72°C for 30 s, and 85°C for 15 s with a fluorescence reading at the last step. A finial cycle was performed with 94°C for 20 s, 55°C for 30 s and 72°C for 180 s, followed by melt curve analysis which was performed from 50-99°C in increments of 1.0°C each step. The qPCR conditions for *Nitrospira*-like NOB *nxrB* gene and comammox *Nitrospira* Clade B *amoA* genes were the same except that the annealing temperature was 60°C. Conditions for qPCR of bacterial 16s rRNA were 94°C for 2 mins, followed by 40 cycles of 94°C for 10 s, 56°C for 30 s with a fluorescence reading at this step. Melt curve analyses was also performed to confirmed the consistency of 16s rRNA amplification. Standard curves were generated using 10-fold dilutions of standards that were made using the same primer pairs used for qPCR.

# Supplementary Tables

**Table S1** Chemical properties of the soil used in the microcosm study.

| **Soil Analysis** | **Value** | **Soil Analysis** | **Value** |
| --- | --- | --- | --- |
| pH | 6.0 | Organic C | 48.0 g kg ^-1^ |
| Exchangeable Ca^+^ | 6.30 cmol_c_ kg ^-1^ | Olsen-P | 45.60 mg kg^-1^ |
| Exchangeable Mg^2+^ | 0.98 cmol_c_ kg ^-1^ | CEC | 15.0 cmol_c_ kg ^-1^ |
| Exchangeable K^+^ | 0.59 cmol_c_ kg ^-1^ | Total N | 2.50 g kg^-1^ |
| Exchangeable Na^+^ | 0.29 cmol_c_ kg ^-1^ | Organic S | 19.0 mg kg^-1^ |
|  |  |  |  |

Core Length = 0-7.5 cm soil

**Table S2** Primers used for qPCR and high-throughput sequencing amplifications.

| **Target** | **Primer** | **Sequence (5' to 3')** | **Reference** |
| --- | --- | --- | --- |
| Bacterial 16S rRNA gene | 1369F | CGGTGAATACGTTCYCGG | Suzuki *et al*., 2000 |
|  | 1492R | GGWTACCTTGTTACGACTT |  |
|  |  |  |  |
| AOA *amoA* | Arch-amoAF | STAATGGTCTGGCTTAGACG | Francis *et al*., 2005 |
|  | Arch-amoAR | GCGGCCATCCATCTGTATGT |  |
|  |  |  |  |
| AOB *amoA* | amoA1F | GGGGTTTCTACTGGTGGT | Rotthauwe et al., 1997 |
|  | amoA2R | CCCCTCKGSAAAGCCTTCTTC |  |
|  |  |  |  |
| Comammox *Nitrospira* Clade A *amoA* | CA377f | GTGGTGGTGGTCBAAYTA | Jiang *et al*., 2020 |
|  | C576r | GAAGCCCATRTARTCNGCC |  |
|  |  |  |  |
| Comammox *Nitrospira* Clade B *amoA* | CB377f | GTACTGGTGGGCBAAYTT | Jiang *et al*., 2020 |
|  | C576r | GAAGCCCATRTARTCNGCC |  |
|  |  |  |  |
| Comammox *Nitrospira* Clade A *amoA* | comaA-244f | TAYAAYTGGGTSAAYTA | Pjevac et al., 2017 |
|  | comaA-659r | ARATCATSGTGCTRTG |  |
|  |  |  |  |
| Comammox *Nitrospira* Clade B *amoA* | comaB-244f | TAYTTCTGGACRTTYTA | Pjevac et al., 2017 |
|  | comaB-659r | ARATCCARACDGTGTG |  |
|  |  |  |  |
| *Nitrospira*-like NOB (All *Nitrospira* lineages includes comammox) | nxrB169F | TACATGTGGTGGAACA | Pester *et al*., 2014 |
|  | nxrB638R | CGGTTCTGGTCRATCA |  |
|  |  |  |  |
| sNOB *cynS* | Ntspa-cynSF | TSATCGGHGTSTAYGGMGA | Jiang *et al*., 2020 |
|  | Ntspa-cynSR | CCGTTCARSGTRATCTTGCA |  |
|  |  |  |  |
| 1^st^ round nested-PCR | A189Y | GGNGACTGGGAYTTYTGG | Xia *et al*., 2018 |
| 2^nd^ round nested-PCR | CA209f | GAYTGGAARGAYCGNCA |  |
| C576r-barcode | A2-C576r | **TTAGGCTAGCTT**GAAGCCCATRTARTCNGCC | This study |
|  | B2-C576r | **TAGCTTCGATGT**GAAGCCCATRTARTCNGCC | This study |
|  | C2-C576r | **ACTTGACTTGTA**GAAGCCCATRTARTCNGCC | This study |
|  | D2-C5762 | **ATTCCTCAGATC**GAAGCCCATRTARTCNGCC | This study |
|  | E2-C5762 | **GCGGACATAGGT**GAAGCCCATRTARTCNGCC | This study |
|  |  |  |  |

**Table S3** Nitrogen dynamics, ammonium-N and nitrate-N, of the 94-days microcosm study.

|  | **NH4^+^-N (mg kg^–1^ dry soil)** | | | | | | |
| --- | --- | --- | --- | --- | --- | --- | --- |
| **Sample** | **Day 1** | **Day 7** | **Day 15** | **Day 44** | **Day 58** | **Day 80** | **Day 94** |
| Control | 1.68 ± 0.27 | 4.82 ± 0.53 | 4.82 ± 1.73 | 1.62 ± 0.16 | 2.17 ± 0.48 | 1.35 ± 0.41 | 1.74 ± 0.34 |
| FDE | 2.70 ± 0.58 | 8.20 ± 2.79 | 6.03 ± 2.56 | 1.62 ± 0.10 | 2.15 ± 0.45 | 0.82 ± 0.26 | 2.32 ± 0.43 |
| S700 | 699.14± 14.46 | 768.96 ± 7.03 | 629.11 ± 23.33 | 393.30 ± 7.93 | 225.53 ± 8.38 | 155.47 ± 11.18 | 125.40 ± 4.13 |
| S700+DCD | 674.69 ± 45.30 | 771.30 ± 7.46 | 709.87 ± 9.79 | 642.06 ± 11.19 | 570.11 ± 7.27 | 332.27 ± 22.27 | 316.95 ± 35.17 |
| U50 | 42.55 ± 2.09 | 25.46 ± 2.69 | 4.70 ± 0.62 | 2.09 ± 0.08 | 1.91 ± 0.46 | 1.15 ± 0.26 | 1.64 ± 0.49 |
| U50+DCD | 47.12 ± 2.13 | 53.06 ± 3.99 | 57.52 ± 2.68 | 53.66 ± 4.61 | 58.53 ± 4.46 | 19.50 ± 3.55 | 3.67 ± 0.83 |
|  |  |  |  |  |  |  |  |
|  | **NO_3_^–^-N (mg kg^–1^ dry soil)** | | | | | | |
| **Sample** | **Day 1** | **Day 7** | **Day 15** | **Day 44** | **Day 58** | **Day 80** | **Day 94** |
| Control | 10.77 ± 0.32 | 18.18 ± 0.77 | 26.21 ± 1.93 | 42.64 ± 3.14 | 47.93 ± 4.63 | 57.24 ± 4.54 | 66.96 ± 7.63 |
| FDE | 13.08 ± 0.50 | 21.84 ± 1.42 | 34.33 ± 3.42 | 42.61 ± 3.72 | 57.85 ± 0.95 | 59.33 ± 0.65 | 95.21 ± 22.45 |
| S700 | 10.57 ± 0.67 | 32.84 ± 4.56 | 110.36 ± 8.57 | 388.29 ± 24.54 | 457.68 ± 40.90 | 506.49 ± 51.19 | 744.51 ± 36.01 |
| S700+DCD | 9.45 ± 0.45 | 17.61 ± 1.09 | 38.27 ± 0.73 | 87.38 ± 6.79 | 136.73 ± 5.57 | 253.58 ± 10.94 | 536.64 ± 91.72 |
| U50 | 16.54 ± 0.42 | 46.75 ± 1.69 | 71.46 ± 3.22 | 73.80 ± 6.03 | 87.94 ± 4.03 | 88.57 ± 10.19 | 101.56 ± 1.68 |
| U50+DCD | 12.22 ± 0.40 | 20.46 ± 1.33 | 29.25 ± 1.46 | 38.13 ± 2.95 | 58.57 ± 2.28 | 84.78 ± 2.64 | 114.53 ± 3.77 |
|  |  |  |  |  |  |  |  |

**Table S4** Illumina NovaSeq sequencing data quality summery of *amoA* gene from the 94-day microcosm study.

| **Sample** | **Raw reads** | **Raw data (Gb)** | **Effective (%)** | **Error (%)** | **Q20 (%)** | **Q30 (%)** | **Average GC content (%)** | **Final reads** |
| --- | --- | --- | --- | --- | --- | --- | --- | --- |
| Control | 7051482 | 2.3 | 98.65 | 0.04 | 94.35 | 87.11 | 51.85 | 956450 |
| U50 | 7328460 | 1.8 | 99.05 | 0.03 | 96.51 | 90.76 | 51.99 | 925178 |
| U50 + DCD | 7377506 | 1.8 | 99.16 | 0.03 | 96.43 | 90.62 | 52.18 | 886421 |
| FDE | 8068196 | 2.1 | 99.19 | 0.03 | 96.86 | 91.37 | 51.55 | 876982 |
| S700 | 7753340 | 2.5 | 99.10 | 0.03 | 94.86 | 87.95 | 54.78 | 981016 |
| S700 + DCD | 7054216 | 1.8 | 99.09 | 0.03 | 96.03 | 90.35 | 54.34 | 882425 |

Raw reads =total number of reads of paired─end sequencies, read 1 and read 2 combined
Raw data = raw reads × sequence length
Effective = (clean reads/raw reads)×100%
Error = base error rate
Q20(%) or Q30(%) = base count of Phred value > 20 or 30 / total number of bases
Average GC content = number of G & C base / total number of bases
Final reads = total number of reads after *in-silico* bioinformatics pipeline outlined in Section 2.7

# Supplementary Figures


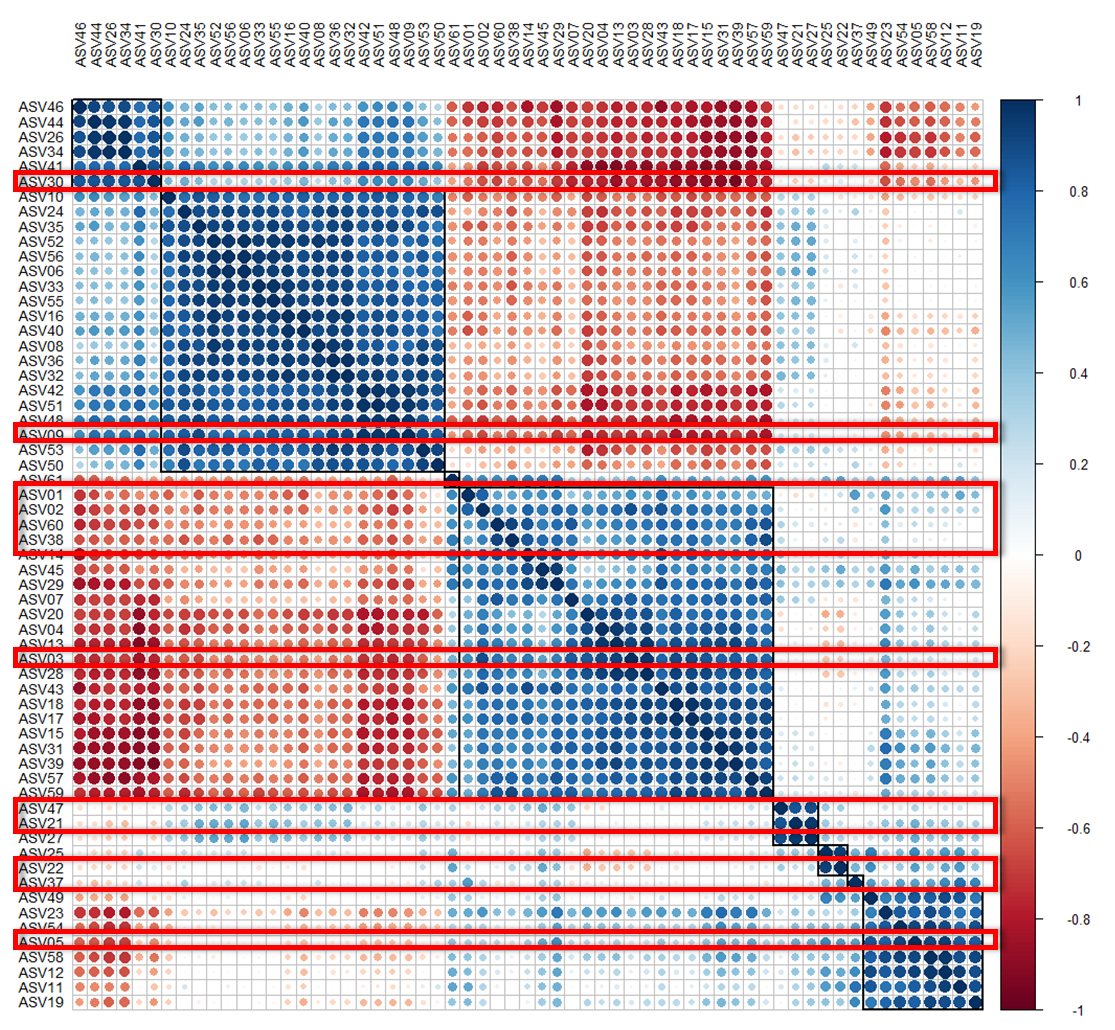


Fig. S1 Correlations of all amplicon sequence variant obtained. Correlation heatmap plot exhibits the pairwise Spearman’s correlations between each ASVs and their relative abundance in all treatments. Black rectangles around the correlation matrix are based on the results of hierarchical clustering. Scale bars indicate correlation coefficients. Red box indicating variant that has been chosen for downstream analysis.


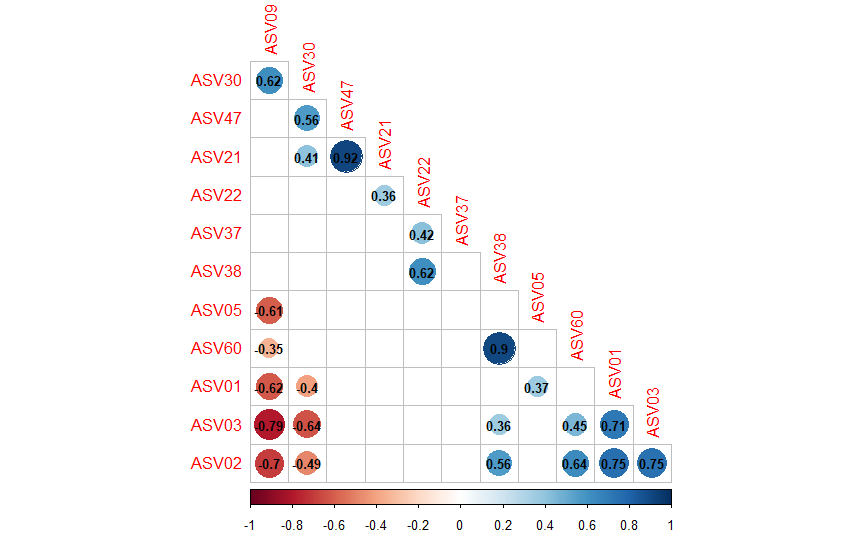


Fig. S2 Spearman correlation of relative abundance of 12 selected ASVs. Pairwise comparisons of 12 selected ASVs community composition using spearman correlation test which the color gradient represents correlation coefficients. Numbers indicating the spearman’s r value and a value is only shown when the correlation coefficients are significant (*p* < 0.001).


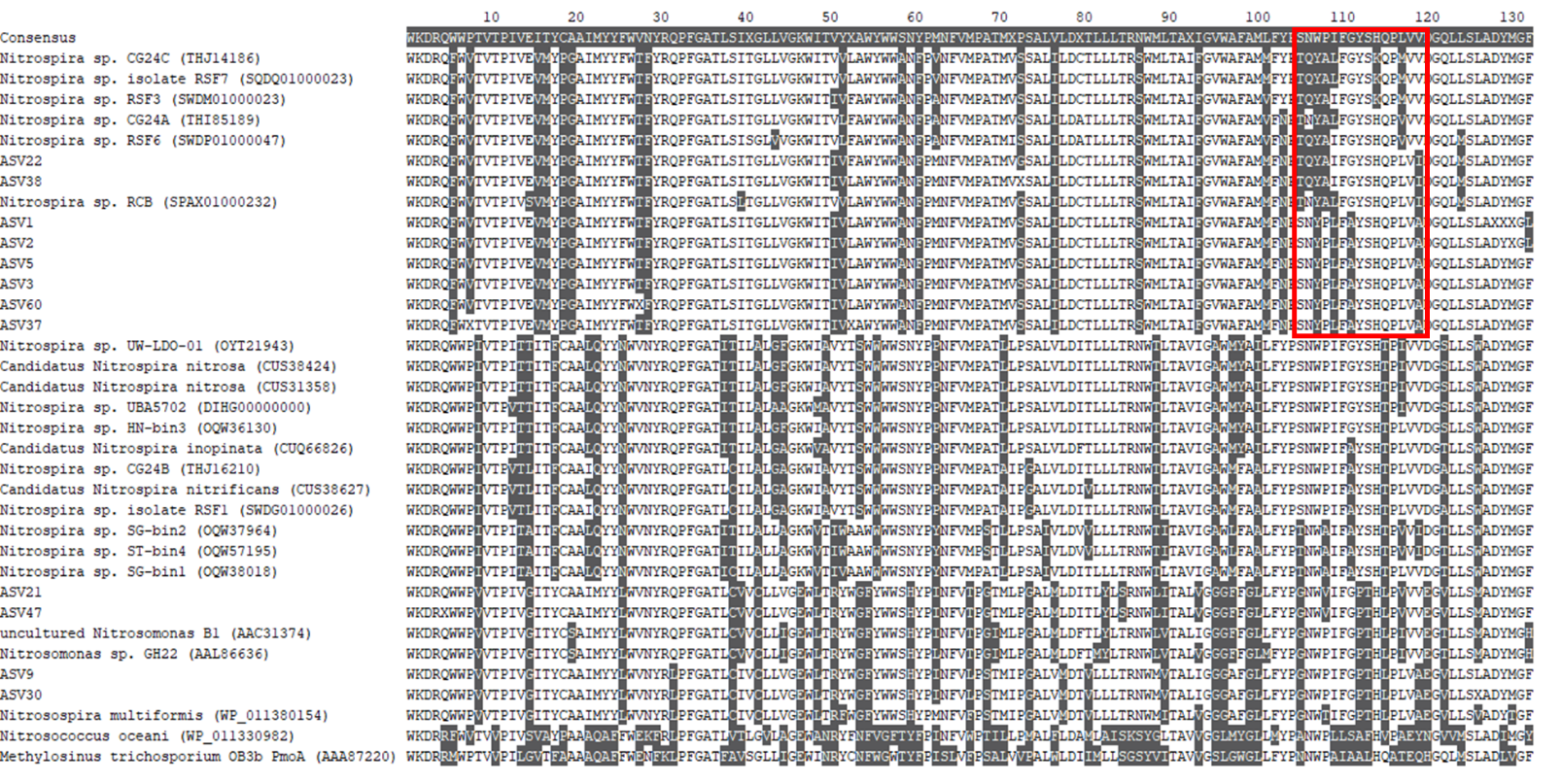


Fig. S3 Comammox *Nitrospira*, *Nitrosomoans* spp., and *Nitrosospira* spp. AmoA protein sequence alignment. AmoA protein sequence alignment of 133 amino acids that resembles residues 58–190 of *Candidatus Nitrospira inopinata* AmoA protein (CUQ66826). Red box highlighted the most diverse region between comammox *Nitrospira* Clade B1 and B2, which resides from residues 74–126.


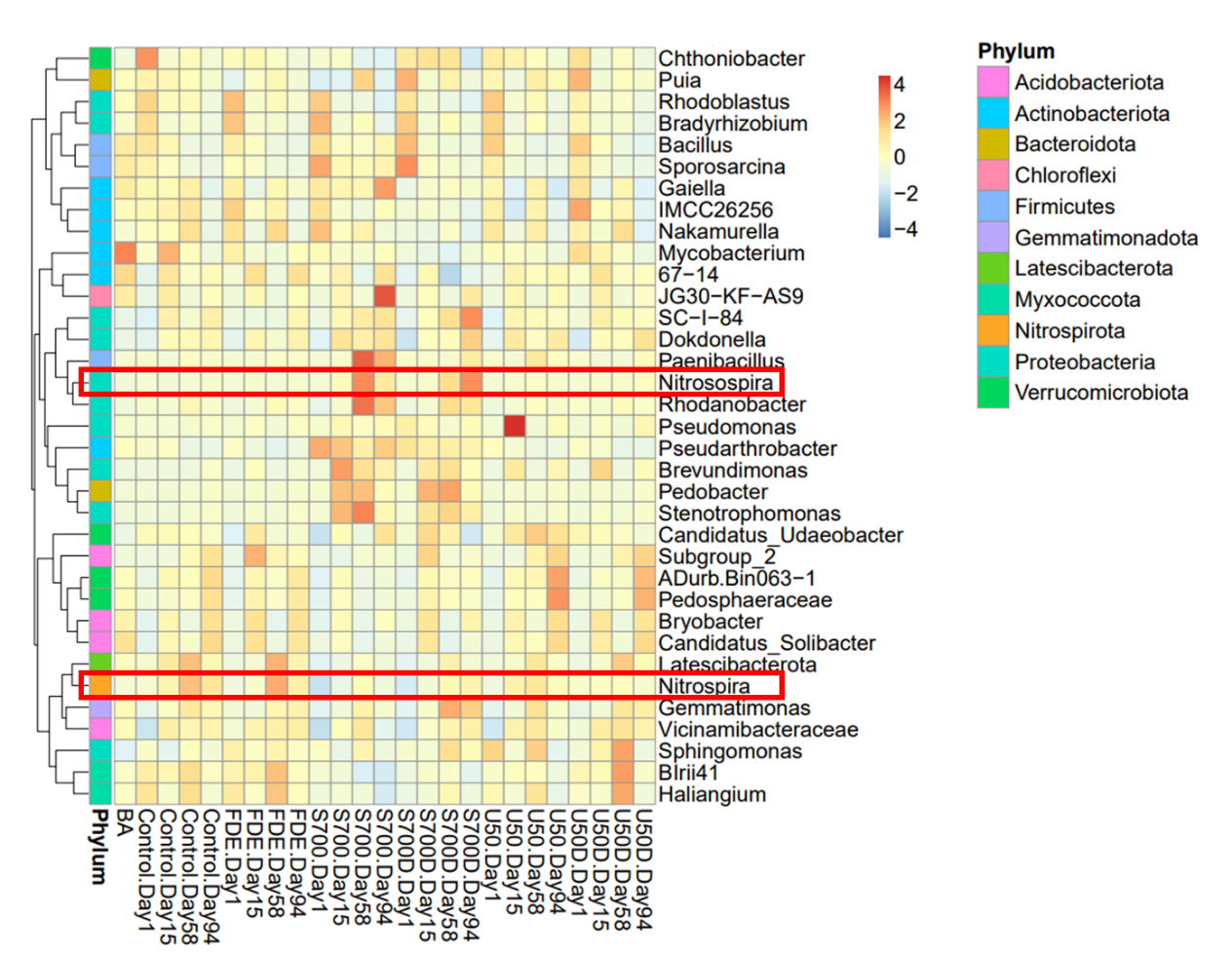


Fig. S4 Structural differences in microbial communities. Heatmap of the bacterial community composition relative to abundance of microbial taxa characterized by 16S rRNA gene sequencing of the most abundant amplicon sequence variants (ASVs) across treatments. The plot is ordered by distance-based clustering (Euclidian) of the top 10 most abundant ASVs for each treatment at Day 1, 15, 58 and 94. Gene relative abundances (4 to -4) are indicated via color-coding from red to blue. Phylum-level are indicated in different colors and genus-level is indicated on the right-hand side. Red box highlighted relative abundance of *Nitrosospira* (AOB) and *Nitrospira* (comammox + *Nitrospira*-like NOB).


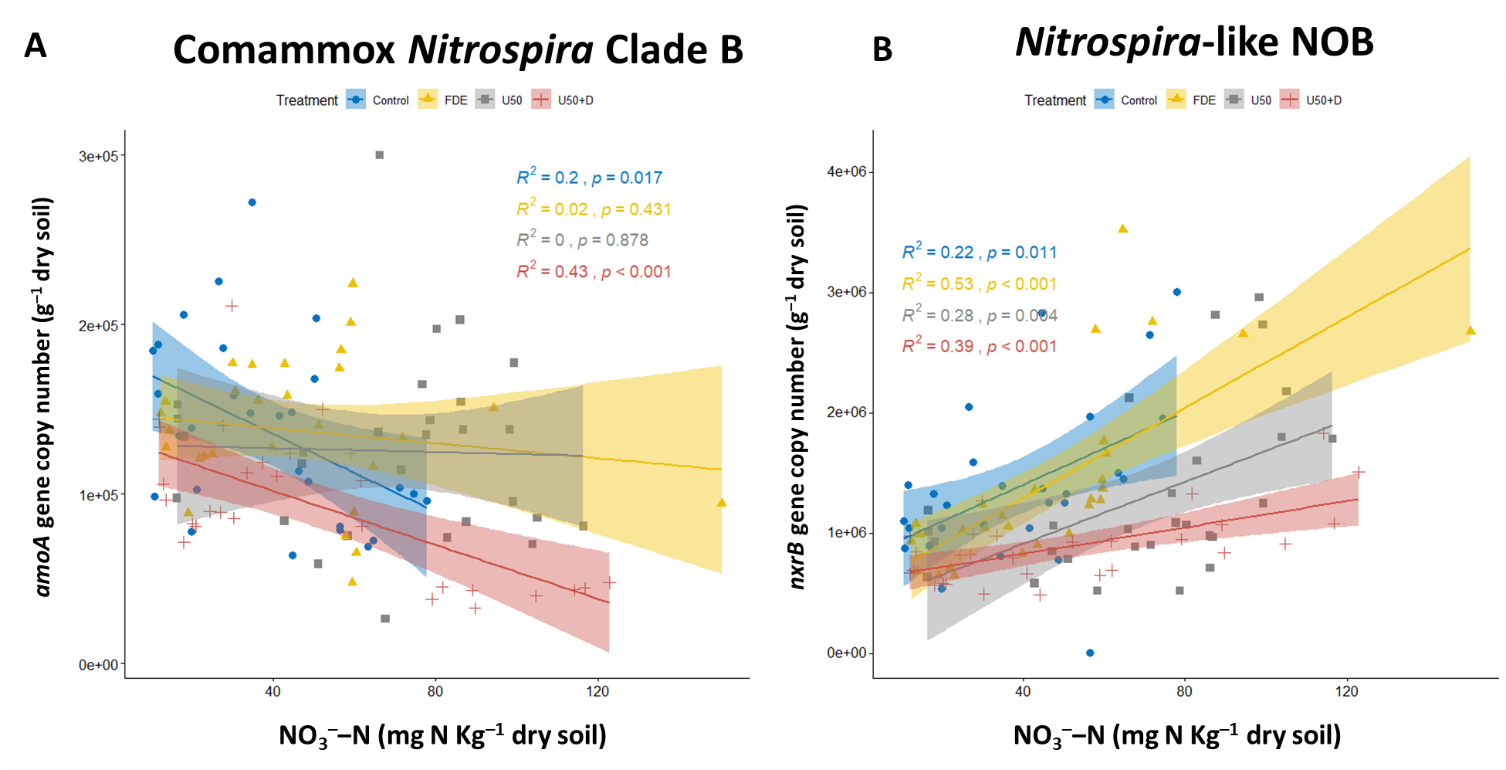


Fig. S5 Correlation between nitrate and *Nitrospira* lineages. Linear regression between nitrate–N and comammox *Nitrospira* Clade B *amoA* (A) and *Nitrospira*-like NOB *nxrB* gene copy numbers (B). Color solid lines indicates the ordinary least square liner regressions. Shaded area represents 95% confidence intervals. Coefficient of determination R^2^ and *p* values of each treatment are stated.
